# Supplementary material for: System wide analyses have underestimated protein abundances and the importance of transcription in mammals
Source: PeerJ. 2014 Feb 27;2:e270. doi: 10.7717/peerj.270 (PMC3940484; doi:10.7717/peerj.270)
Supplement: Supplemental Information 7 [file peerj-02-270-s007.pdf]

Table S1. Variances in translation rates estimates

Cell line	Dataset	# genes	variance*
NIH3T3, mouse	Inferred, Schwanhausser all[a]	3,633	0.2872103
NIH3T3, mouse	Inferred, Schwanhausser intersect[b]	3,126	0.2739192
NIH3T3, mouse	Ribo FP, Subtelny[c]	6,012	0.04070817
NIH3T3, mouse	Ribo FP, Subtelny intersect[d]	3,126	0.03374892
Neutrophil, mouse	Ribo FP, Guo[e]	5,796	0.04145488
HeLa, mouse	Ribo FP, Guo[e]	5,742	0.08086076
ES, mouse	Ribo FP, Ingolia[f]	10,217	0.06213300

* Replica data is available for all genes used for the NIH3T3 datasets. In these cases, the means of each pair of measurements was calculated and the variance of these means is given. The data for Schwanhausser et al. is from Dataset S1. The data for Subtelny et al. is the miR-155 and miR-1 NIH3T3 cell line data from GEO GSE52809. For other cell lines only a single dataset is available, and thus the variance in the single gene measurements was determined.

[a] Data from Schwanhausser et al., 2011 for which translation rates were determined for both replicas.

[b] Data from Schwanhausser et al., 2011 for the intersection of genes in dataset a. and all genes detected by Subtelny et al.

[c] Data from Subtelny et al., 2014 for genes detected at >= 10 RPKM for mRNA seq and ribosome footprints.

[d] Data from Subtelny et al., 2014 for the intersection of genes in dataset a. and all genes detected by Subtelny et al.

[e] Data from Guo et al., 2010 for all genes detected at >100 reads in both mRNA-seq and ribosome footprints.

[f] Data from Ingolia et al., 2011 for all genes reported.
